# Contribution of psychosocial factors to socioeconomic inequalities in mortality among older Australian men: a population-based cohort study

**DOI:** 10.1186/s12939-020-01277-2

**Published:** 2020-10-07

**Authors:** Saman Khalatbari-Soltani, Fiona Stanaway, Erin Cvejic, Fiona M. Blyth, Vasi Naganathan, David J. Handelsman, David G. Le Couteur, Markus J. Seibel, Louise M. Waite, Robert G. Cumming

**Affiliations:** 1 The University of Sydney School of Public Health, Faculty of Medicine and Health, Sydney, New South Wales Australia; 2 ARC Centre of Excellence in Population Ageing Research (CEPAR), University of Sydney, Sydney, Australia; 3 Concord Clinical School, Faculty of Medicine and Health, University of Sydney, Sydney, New South Wales Australia; 4 Centre for Education and Research on Ageing, Faculty of Medicine and Health, University of Sydney, Sydney, New South Wales Australia; 5 Ageing and Alzheimer’s Institute, Concord Repatriation and General Hospital, Sydney Local Health District, Sydney, New South Wales Australia; 6 ANZAC Research Institute, University of Sydney and Concord Hospital, Sydney, Australia

**Keywords:** Socioeconomic status, Psychosocial factors, Social support, Psychological, distress, Mortality, Older adults

## Abstract

**Background:**

Among older people, the extent to which psychosocial factors explain socioeconomic inequalities in mortality is debated. We aimed to investigate the potential mediating effect of psychosocial factors on socioeconomic inequalities in mortality.

**Methods:**

We used data from a prospective population-based cohort (the Concord Health and Ageing in Men Project; baseline recruitment in 2005–2007), in Sydney, Australia. The main outcomes were all-cause and cause-specific mortality. Socioeconomic status (SES; educational attainment, occupational position, source of income, housing tenure, and a cumulative SES score) was assessed at baseline. Measures of structural and functional social support, as well as depressive and anxiety symptoms were assessed three times during follow-ups. Associations were quantified using Cox regression. Mediation was calculated using “change-in-estimate method”.

**Results:**

1522 men (mean age at baseline: 77·4 ± 5·5 years) were included in the analyses with a mean (SD) follow-up time of 9·0 (3·6) years for all-cause and 8·0 (2·8) years for cause-specific mortality. At baseline, psychosocial measures displayed marked social patterning. Being unmarried, living alone, low social interactions, and elevated depressive symptoms were associated with higher risk of all-cause and cardiovascular disease (CVD) mortality. Psychosocial factors explained 35% of SES inequalities in all-cause mortality, 29% in CVD mortality, 12% in cancer mortality, and 39% in non-CVD, non-cancer mortality.

**Conclusion:**

Psychosocial factors may account for up to one-third of SES inequalities in deaths from all and specific causes (except cancer mortality). Our findings suggest that interventional studies targeting social relationships and/or psychological distress in older men aiming to reduce socioeconomic inequalities in mortality are warranted.

## Introduction

Previous research has demonstrated that socioeconomic inequalities in mortality from all and specific causes persist into older age [1, 2]. Socioeconomic inequalities in mortality are a major public health issue and quantifying modifiable intermediate factors in the link between low socioeconomic status (SES) and increased mortality risk has important implications for health and social policy [2, 3].

It is well established that structural and functional social support and psychological distress are related to an increased risk of mortality [4–6]. In addition, individuals with low SES are more likely to have low levels of social support, to be less socially integrated [7, 8], and have higher risk of anxiety and depressive symptoms [9, 10]. This has led researchers to hypothesize that social relationships and psychological distress could be one mechanism underlying socioeconomic inequalities in mortality [11]. A small number of studies have investigated the mediating role of social support and psychological distress on SES inequalities in mortality [8, 12–17]. However, most commonly these studies relied on a single indicator of SES [8, 12–16], examining only all-cause mortality [12, 16, 17] and/or CVD-mortality [8, 13–15]. Moreover, only three of these studies were among middle-aged and older individuals [8, 13, 16], and the rest were among individuals with a wide range of age distributions (15 to 80 years) without analyses stratified by age [12, 14, 15, 17]. So, the mediating role of social support and psychological distress on SES inequalities in mortality from all and specific causes at older ages, when the burden of mortality is at its greatest, remain unknown.

Given the limited evidence, we aimed to investigate the potential mediating effect of social relationships and psychological distress in the association between SES and mortality. To do this, we investigated the association of SES with social relationships and psychological distress, as well as the extent to which social relationships and psychological distress are associated with mortality among older adults. To provide a broader perspective of these associations we examined both all-cause and cause-specific mortality. As to date epidemiological studies of ageing have tended to focus on women, and as some previous studies reported that socioeconomic inequalities in mortality were more pronounced among men [18], we used data from a population-based cohort of older Australian men.

## Methods

### Study population

We used data from an on-going population-based cohort study, the Concord Health and Ageing in Men project (CHAMP) [19]. CHAMP recruited 1705 men aged ≥70 years (2005–2007) in a defined geographical region in the city of Sydney, Australia, through the New South Wales Electoral Roll, on which registration is compulsory for Australian citizens, making it a suitable population-wide sampling frame. The only exclusion criteria of the CHAMP study was living in an aged care facility. Eligible men were sent an invitation letter describing the study (*n* = 3627) and, if they had a listed telephone number, were telephoned about one week later (*n* = 3005). Men without listed telephone numbers who did not respond to the first letter were sent a second invitation letter (*n* = 622). Among contacted men, 190 were not eligible as they had moved out of the study area, moved into nursing home, or had died. Of the 2815 eligible men with whom contact was made, 1511 participated in the study (54%); lack of time and interest, as well as health problems were the main reasons for non-participation. An additional 194 men living in the study area heard about the study from friends or the local media and were volunteered to be in the study before receiving a letter, yielding a total cohort of 1705 participants.

Participating men underwent baseline assessments which comprised self-completed questionnaire, interview-administered questionnaires, and a wide range of clinical assessment. Data were collected by fully trained staff.

There were three further study phases: the first follow-up in 2007–2009 (*n* = 1366; 85% of living men), the second follow-up in 2012–2013 (*n* = 954; 72% of living men), and the third follow-up in 2015–2016 (*n* = 779; 71% of living men) [19]. Death and illness were the two main reasons for non-participation at follow-up study waves. The CHAMP study complied with the World Medical Association Declaration of Helsinki and was approved by the Sydney South West Area Health Service Human Research Ethics Committee. Written informed consent was obtained from all participants.

### Socioeconomic indicators

Baseline self-reported educational attainment, occupational position, sources of income, and housing tenure were used as individual indicators of SES. Highest educational qualification was grouped into ‘high’ (university degree), ‘intermediate’ (trade, apprenticeship, certificate, or diploma), and ‘low’ (no post-school qualification). Occupational position based on longest occupation held during working life was categorized into ‘high’ (higher professional and managers, lower professionals and managers, higher clerical services and sales workers), ‘medium’ (small employers and self-employed, farmers, lower supervisors and technicians), and ‘low’ (lower clerical, services, sales workers, skilled and unskilled workers) [20]. Source of income was categorized into ‘high’ (sources of income do not include any government pension), ‘intermediate’ (reliant on a government pension *plus* other sources of income), and ‘low’ (reliant solely on a government pension). Australia’s retirement income system comprises a means-tested age pension, mandatory occupational superannuation, and voluntary long-term savings [21]. Housing tenure was categorized as ‘owner’ (owning home outright), and ‘other’ (e.g. leasing or purchasing in a retirement village, paying rent to a private landlord, and paying rent to the government for public housing).

A cumulative SES score representing cumulative exposure to low SES from early adult life to older ages was calculated using the four individual indicators of SES at baseline, as previously conducted [13]. Educational attainment, occupational position, and source of income were coded 0–2; housing tenure was coded 0 (owners) or 1 (other). The four SES indicators were summed, resulting in a 7-level cumulative SES score with higher values corresponding to greater disadvantage.

### Measures of structural and functional social support

We used longitudinal measures of marital status, living arrangements, family and non-family social network size, and social interaction score as measures of structural social support. Marital status was divided into married/defacto and single or divorced/widowed. Two separate living arrangement variables were used: living alone (yes/no) and living with children or grandchildren (yes/no). Family and non-family network size were obtained from a modified question in the Duke Social Support Index (DSSI), “How many persons within one hour travel of your home do you feel you can depend on or feel very close to?”. We then created two dichotomous variables: family and non-family support (yes = having one or more persons, no = having no one).

Social interaction score was based on three items of the DSSI about the number of times spent with someone that the participant does not live with, the number of times the participant talked to someone on the telephone, and the number of times the participant attended meetings of social clubs, religious meetings or other groups within the past week. Each item had eight frequency options from “none” to “seven or more”. A score of one was assigned to “none”, two points to “once or twice”, and three points to “three times or more”. The resulting social interaction score ranged between 3 to 9 which was then dichotomized at the lowest quartile (≤5).

We used longitudinal measures of social satisfaction as a measure of functional social support. Social satisfaction score was based on 7 items in the DSSI; 6 items covered participants’ involvement in relationships and perceived availability and adequacy of relationships with three possible answers: “hardly ever” (score 1), “some of the time” (score 2), or “most of the time” (score 3). The final item measures participants’ satisfaction with their relationship with family or friends with three possible answers: “very satisfied” (score 1), “somewhat satisfied” (score 2), or “satisfied” (score 3). The resulting social satisfaction score ranged between 0 to 21 which was then dichotomized at the lowest quartile (≤19).

### Psychological distress

We used psychological distress data from baseline and follow-ups of the study. Depressive symptoms were measured using the validated short version of the self-completed Geriatric Depression Scale (GDS, ranges between 0 to 15) [22]. Elevated depressive symptoms was defined by GDS score ≥ 5 [23]. Anxiety symptoms were measured using the validated self-completed Goldberg Anxiety Scale (GAS, ranges between 0 to 9) [24]. Participants with a GAS score ≥ 5 were categorized as having clinically elevated anxiety symptoms [24].

### Mortality ascertainment

Consenting participants (*n* = 1639, 96%) were linked to the New South Wales Registry of Births, Deaths, and Marriages (RBDM; records all deaths in New South Wales), by the Centre for Health Record Linkage (http://www.cherel.org.au/) using probabilistic record linkage methods and Choice-Maker software. Mortality follow-up was available up to December 31, 2017 for all-cause mortality and up to December 31, 2015 for cause of death. Deaths from all-cause, cardiovascular disease (CVD; ICD-10 codes I00-I99), cancer (ICD-10 codes C00-C97), and non-CVD, non-cancer were examined.

### Assessment of covariates

We included baseline age (continuous), age squared (continuous), and country of birth (Australian-born/other), as well as longitudinal measures of health-related behaviours (alcohol consumption, smoking, and physical activity), body mass index (BMI; weight divided by height squared-kg/m^2^), and self-rated health as potential confounders. Alcohol consumption was categorized as ‘abstainer’ (0 unit/week during the past year), ‘moderate drinker’ (1–21 units/week), or ‘heavy drinker’ (> 21 units/week) based on the number of alcohol units consumed in the past year. Smoking was categorized as ‘never smoker’, ‘former smoker’, and ‘current smoker’. The Physical Activity Scale for the Elderly (PASE) was used to measure physical activity and the score was dichotomized at the lowest quartile (< 79 vs ≥80) as the distribution was highly positively skewed. BMI was categorized as normal or underweight (BMI < 25 kg/m^2^), overweight (25 ≤ BMI < 30 kg/m^2^), and obese (BMI ≥ 30 kg/m^2^). Self-rated health was measured using the single question “Compared to other people your own age, how would you rate your overall health?”. Responses were dichotomized into “excellent/good” and “fair/poor/very poor”.

### Statistical analysis

Statistical analyses were performed using Stata (version 15; StataCorp, College Station, TX, USA). We accounted for baseline missing values for health-related behaviours (*n* = 52) and missing values for social relationships, psychological distress, and health-related behaviours at follow-ups, using chained equations (see Supplementary Table 1) [25]. Ten imputed datasets were generated and analysed. The imputation model included age, all confounding variables, mediating variables, and survival status [26]. Missing data on marital status were replaced with data from the previous follow-up.

Cross-sectional associations of baseline individual and cumulative SES indicators with social relationships and psychological distress were assessed by multivariable logistic regression. We ran three sets of models adjusted for potential confounders based on previously observed associations in earlier high-quality studies [8, 27]. Model 1 was adjusted for age, age squared, and country of birth. In model 2, we additionally adjusted for baseline health-related behaviours and BMI. Model 3 was additionally adjusted for baseline self-rated health.

We examined the associations of baseline social relationships and psychological measures with mortality endpoints using Cox proportional-hazards regression. Associations with cause-specific mortality were examined using Fine and Gray’s competing-risks survival regression (proportional sub-hazards model) [28]. We ran the same three sets of models as described in the previous paragraph. Adjusted hazard ratios (HRs) or sub-hazard ratios (SHRs) and 95% confidence intervals (CIs) are reported.

Cox proportional-hazards regression was also used to assess the associations between baseline SES indicators and all-cause mortality. Competing-risk survival regression was used for cause-specific mortality. Models were adjusted for age, age squared, and country of birth (reference model). HRs and SHRs were calculated for the most disadvantaged versus least disadvantaged groups. Survival time was measured as the time from the date of baseline interview to either the date of death or end of follow-up (December 31, 2017 for all-cause mortality; December 31, 2015 for cause-specific mortality). The proportional hazards assumption was assessed using Schoenfeld residuals; in all models this assumption was satisfied. Preliminary analyses showed no interaction between social relationships, psychological measures, and SES indicators with age group (70–70 and ≥ 80 years) and country of birth (p_interaction_ > 0.05).

To assess the extent to which longitudinally assessed structural and functional social support and psychological distress explained SES inequalities in mortality, we calculated the percentage attenuation using the “change-in-estimate” method for the following groups of explanatory variables: 1) structural social support (marital status, live with children, family and non-family support, and social interactions score); 2) functional social support (social satisfaction score); and 3) psychological distress (depressive and anxiety symptoms). As living alone and marital status were highly correlated (Spearman r = 0.78) we did not include living alone in our model; collinearity among other psychosocial measures was low (Spearman r ≤ 0.32). There were no interactions between SES indicators and psychosocial measures (all P_interaction_ > 0·05). For each risk-factor group, we calculated the attenuation percentage as 100 * (β_Model 1_- β_Model1 + psychosocial measures(s)_)/ (β_Model 1_), as previously conducted [29].

### Sensitivity analyses

To investigate the potential influence of reverse causality, that is, baseline presence of diagnosed and undiagnosed comorbid conditions (raising the short-term risk of mortality), which may influence SES, we repeated the analyses after excluding participants who died during the two first years of follow-ups. To assess robustness of results, we repeated our analyses using data from those participants who did not have any missing data (complete-case analysis).

## Results

Out of 1705 participants at baseline, 183 (10·7%) were excluded due to inability to link to mortality data or missing socioeconomic or psychosocial measures at baseline, leaving 1522 participants for analysis (see Supplementary Figure 1). Excluded participants were more likely to be overseas-born and live with their children (see Supplementary Table 2). The mean age of study participants was 77·4 (SD 5·5) years. Majority of participants were aged less than 80 years and married. Less than 20% of participants lived alone, have no family and non-family support, and have elevated depressive symptoms. Majority of participants were moderate alcohol drinkers and physically active. In comparison to participants in high SES group, those in the low SES group (cumulative SES score 5–7) tended to be younger (75·2% vs. 69·8%) and were more likely to be overseas-born (71·4% vs. 34·6%), to live with their children (25·7% vs. 13·4%), to have no non-family support in the area (33·2% vs. 18·1%), to have lower social interactions and satisfaction scores, to have elevated depressive symptoms (23·2% vs. 9·1%), and to have fair, poor or very poor self-rated health (38·4% vs. 25·3%) (Table 1). Those in the low SES group were also more likely to be a current smoker (10·2% vs. 3·4%) and be physically inactive (28·5% vs. 22·5%) but were less likely to be heavy alcohol drinkers (6·8% vs. 8·6%) than those in high SES group (Table 1). Baseline characteristics of participants by individual indicators of SES are shown in Supplementary Table 3.

**Table 1:** Baseline characteristics of participants by cumulative socioeconomic status score, the CHAMP study

Characteristics	Overall sample	Tertile groups of cumulative SES		
		High (0–3)	Intermediate (4)	Low (5–7)
N	1522	768	314	440
Age, years	77·4 ± 5·5	77·5 ± 5·7	77·8 ± 5·6	77·1 ± 5·2
Age categories				
70–79 (*n* = 1080)	71·0	69·8	67·8	75·2
80+ (*n* = 442)	29·0	30·2	32·2	24·8
Country of birth				
Australian-born (*n* = 784)	51·5	65·4	49·7	28·6
Other (*n* = 738)	48·5	34·6	50·3	71·4
**Structural and functional social support**				
Marital status				
Married/Defacto (*n* = 1168)	76·7	78·9	73·2	75·5
Not married (*n* = 354)	23·3	21·1	26·8	24·5
Live alone (*n* = 284)	18·6	17·8	17·8	18·6
Live with children (*n* = 279)	18·3	13·4	20·1	25·7
No family support (*n* = 174)	11·4	10·4	12·1	12·7
No non-family support (*n* = 361)	23·7	18·1	24·2	33·2
Social interaction score	6·0 ± 1·3	6·2 ± 1·3	5·9 ± 1·2	5·7 ± 1·4
Social satisfaction score	19·4 ± 2·3	19·7 ± 2·1	19·3 ± 2·4	18·8 ± 2·7
Social satisfaction				
High (*n* = 955)	62·7	67·1	63·7	54·5
Low (*n* = 567)	37·3	32·9	36·3	45·5
**Psychological distress**				
Depressive symptoms (*n* = 222)	14·6	9·1	15·9	23·2
Anxiety symptoms (*n* = 109)	7·2	6·3	8·6	7·7
**Health-related behaviours**				
Alcohol consumption				
Abstainer (*n* = 351)	23·1	20·6	24·2	26·6
Moderate drinkers (*n* = 1032)	67·8	69·5	68·5	64·3
Heavy drinkers (*n* = 117)	6·8	8·6	6·7	6·8
Missing (n = 22)	1·4	1·3	0·6	2·3
Smoking				
Non-smoker (*n* = 564)	37·1	42·5	37·3	27·5
Ex-smoker (*n* = 868)	57·0	54·0	57·3	62·0
Current smoker (*n* = 88)	5·8	3·4	5·4	10·2
Missing (n = 2)	0·1	0·1	0·0	0·2
Physical activity				
Active (*n* = 1147)	75·4	77·4	75·5	71·8
Inactive (*n* = 372)	24·4	22·5	24·2	28·5
Missing (n = 3)	0·2	0·1	0·3	0·2
Body mass index, kg/m^2^	27·8 ± 3·9	27·4 ± 3·8	28·1 ± 3·7	28·3 ± 4·3
Body mass index categories				
Underweight/normal (n = 362)	23·8	26·2	19·4	22·7
Overweight (*n* = 730)	48·0	48·8	51·9	43·6
Obese (*n* = 405)	26·6	23·7	27·1	31·4
Missing (*n* = 25)	1·6	1·3	1·6	2·3
Self-rated health				
Good or excellent (*n* = 1065)	70·0	74·6	71·0	61·1
Fair, poor, very poor (*n* = 454)	29·8	25·3	29·0	38·4
Missing (n = 3)	0·2	0·1	0·0	0·5

*Abbreviation*: *SES* socioeconomic status

Data are mean ± SD for continuous variables or percent for categorical variables, unless otherwise stated

After adjustment for age, country of birth, health-related behaviours, and self-rated health, a lower SES as assessed by cumulative SES score, sources of income, and not owning a house was associated with lower structural and functional social relationships and elevated depressive symptoms but not anxiety symptoms (Table 2 & Supplementary Table 4). Having a low educational level and occupational position was associated with no non-family support, lower social interactions score, and elevated depressive symptoms (see Supplementary Table 4). Our complete-case analysis provided similar associations to analyses using imputed missing data (see Supplementary Table 5).

**Table 2:** Associations of baseline cumulative socioeconomic status score and psychosocial measures, the CHAMP study

	Model 1	Model 2	Model 3
	OR (95% CI) ^a, b^	OR (95% CI) ^a, c^	OR (95% CI) ^a, d^
**Structural social support**			
Not married	1·76 (1·30 to 2·39)	1·75 (1·28 to 2·39)	1·80 (1·32 to 2·47)
Living alone	1·46 (1·05 to 2·02)	1·44 (1·03 to 2·01)	1·48 (1·06 to 2·07)
Live with children	1·75 (1·28 to 2·39)	1·73 (1·26 to 2·38)	1·76 (1·28 to 2·43)
No family support	1·53 (1·04 to 2·26)	1·44 (0·97 to 2·13)	1·39 (0·93 to 2·07)
No non-family support	1·93 (1·45 to 2·57)	1·91 (1·42 to 2·55)	1·84 (1·37 to 2·46)
Social interaction (low vs. high)	1·77 (1·36 to 2·30)	1·65 (1·26 to 2·16)	1·57 (1·20 to 2·06)
**Functional social support**			
Social satisfaction (low vs. high)	1·52 (1·18 to 1·96)	1·47 (1·13 to 1·90)	1·39 (1·07 to 1·80)
**Psychological measures**			
Depressive symptoms (yes vs. no)	2·70 (1·91 to 3·84)	2·41 (1·68 to 3·46)	2·09 (1·43 to 3·07)
Anxiety symptoms (yes vs. no)	1·38 (0·95 to 2·03)	1·32 (0·89 to 1·94)	1·16 (0·78 to 1·73)

N = 1522

Cross-sectional association between baseline cumulative SES and psychosocial measures were assessed by multivariable logistic regression

^a^ Cumulative socioeconomic status was entered as a 3-level categorical variable; the odd ratio of the lowest versus highest cumulative socioeconomic status are reported here

^b^ Adjusted for age, age squared, and country of birth

^c^ Further adjusted for health-related behaviours (alcohol consumption, smoking, and physical activity), and body mass index

^d^ Further adjusted for self-rated health

### Social relationships, psychological distress, and mortality

During a mean (SD) follow-up time of 9·0 (3·6) years, 777 deaths from all causes occurred (see Supplementary Table 6). There were 200 deaths from CVD, 207 from cancer, and 220 from non-CVD, non-cancer causes during a mean 8·0 (SD: 2·8) years of follow-up (see Supplementary Table 6). Diseases of the respiratory system, diseases of the nervous system, and external causes were the most common causes for non-CVD, non-cancer mortality. Overall, those who died tend to be older, Australian-born, with low social support, and elevated depressive and anxiety symptoms (see Supplementary Table 6).

**Fig. 1:**
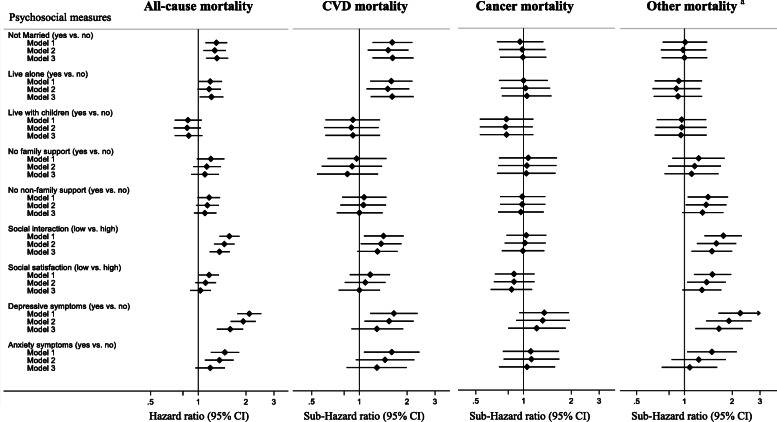
Associations between baseline psychosocial measures and all-cause and cause-specific mortality, the CHAMP study. ^a^ Indicates non-cardiovascular disease and non-cancer mortality. We used calendar year as the time scale, with survivors having a censoring date of 31 December 2017 (person years follow-up = 13,814) for all-cause mortality and with survivors having a censoring date of 31 December 2015 (person years follow-up = 12,180) for cause-specific mortality. Model 1 adjusted for age, age squared, and country of birth. Model 2 further adjusted for health-related behaviours (alcohol consumption, smoking, and physical activity), and body mass index. Model 3 further adjusted self-rated health

Figure 1 shows associations of baseline social relationships and psychological distress with death from all and specific causes. Being unmarried and living alone were associated with higher risk of all-cause and CVD mortality. There were no associations between living with children or not having family and non-family support with mortality. There was a statistically significant association between low social interaction scores and mortality (except cancer mortality) but no associations were evident for low social satisfaction scores. Elevated depressive and anxiety symptoms were associated with all-cause and cause-specific mortality (except for cancer mortality). The associations between elevated anxiety symptoms and mortality were attenuated to the null after adjustment for health-related behaviours and self-rated health. Complete-case analysis provided similar associations to those using imputed missing data (see Supplementary Figure 2).

### Mediating role of social relationships and psychological distress on SES inequalities in mortality

**Fig. 2:**
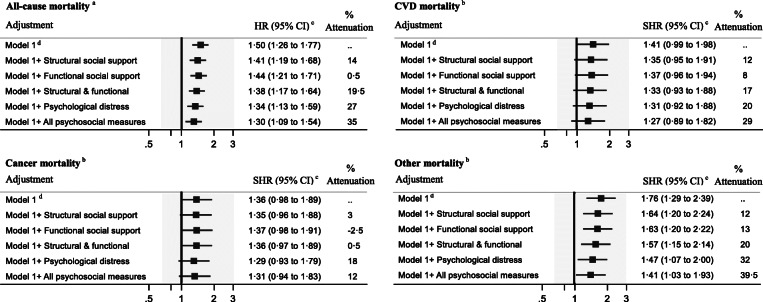
Contribution of longitudinal psychosocial measures in explaining the association between cumulative socioeconomic status score and all-cause and cause-specific mortality, the CHAMP study. Abbreviations: HR, hazard ratio; SHR, sub-hazard ratio. *N* = 1522. There were 777, 200, 207, and 220 deaths attributable to all-cause, CVD, cancer, and non-cancer, non-CVD mortality. ^a^ We used calendar year as the time scale, with survivors having a censoring date of 31 December 2017 (person years follow-up = 13,761). ^b^ We used calendar year as the time scale, with survivors having a censoring date of 31 December 2015 (person years follow-up = 12,126). ^c^ Hazard ratios and sub-hazard ratios for lowest versus highest cumulative socioeconomic status are reported here. Percent attenuation =100 × (β_Model1_ − β_Model1 + psychosocial measures(s)_)/ (β_Model1_), where β = log (Hazard ratio). ^d^ Adjusted for age, age squared, and country of birth. Structural social support: marital status, live with children, family and non-family support, and social interaction score. Functional social support: social satisfaction score. Psychological distress: depressive and anxiety symptoms

For deaths from all causes, the HR for the lowest versus highest cumulative SES groups was 1·50 (95% CI 1·26 to 1·77) in the model adjusted for age, age squared, and country of birth (Fig. 2). The adjusted SHRs were 1·41 (95% CI 0·99 to 1·98) for CVD mortality, 1·36 (95% CI 0·98 to 1·89) for cancer mortality, and 1·76 (95% CI 1·29 to 2·39) for non-CVD, non-cancer mortality (Fig. 2). The associations of cumulative SES with all-cause, and non-CVD, non-cancer mortality remained statistically significant after adjustment for time-varying psychosocial measures. Overall, social relationships and psychological distress combined could account for 35% of the association between cumulative SES score and deaths from all causes; 29% for CVD mortality, 12% for cancer mortality, and 39% for non-CVD, non-cancer mortality (Fig. 2). Psychological distress contributed the most to SES inequalities in mortality (ranging between 18 and 32%). Results of analyses of the mediating role of psychosocial measures in explaining the associations between individual indicators of SES and mortality are presented in Supplementary Table 7.

In sensitivity analyses testing for reverse-causation, the association between baseline SES and mortality remained largely the same after excluding 88 participants who died during the first two years of follow-up (*n* = 1434) (see Supplementary Figure 3). However, the HRs and SHRs were of slightly higher magnitude, particularly for cancer mortality. The mediating role of psychosocial measures was slightly higher than in the whole sample. In the complete-case analysis (*n* = 860), similar effect sizes were evident regarding the association between cumulative SES and mortality from all and specific causes, but CIs were wider (see Supplementary Figure 4). The contribution of psychosocial measures did not substantially differ.

## Discussion

In a representative sample of Australian men aged ≥70 years, we found marital status, living alone, and social interactions, to be associated with all-cause and CVD mortality, but not cancer mortality. Presence of elevated depressive symptoms but not anxiety symptoms was associated with mortality from all and specific causes. Social relationships and psychological distress accounted for more than one-third of SES inequalities in deaths from all-causes, CVD, and non-CVD, non-cancer; and for one-fifth of SES inequalities in cancer mortality. Psychological distress was the most important contributor to SES inequalities in mortality. These observations provide evidence that social relationships and psychological distress might be appropriate targets for public health policies intended to reduce socioeconomic inequalities in mortality of older men.

Our results indicate that being in a low SES group is associated with lower scores for structural and functional social relationships. These associations have been previously reported among older people for education [7, 30, 31], income [7, 30, 31], and occupational position [8, 30]. Moreover, our results show a marked social gradient in depressive symptoms which accords with previous studies [9, 10]. Previous studies have reported direct [9, 32] or null [33] associations between SES indicators and anxiety symptoms among older adults. The null associations reported previously are in line with our results; apart from differences in sample size and anxiety ascertainment, part of the heterogeneity of the results could be due to differences in recognising anxiety compared to depressive symptoms due to low health literacy. Indeed, it has been previously shown that among older adults, inadequate health literacy is associated with lower reporting of anxiety symptoms [34] but not depressive symptoms [35].

### Social relationships, psychological distress, and mortality

In our study, being unmarried and living alone were associated with all-cause and CVD mortality which agrees with previous systematic review of observational studies [36]. In our study, these factors acted independently of health-related behaviours and self-rated health. Protective effects of being married and living with someone increased availability of social and economic support [37].

The association between social interactions and mortality was stronger than the association between social satisfaction and mortality. This could be explained by the fact that social satisfaction reported by the participants was quite high, thus there may be insufficient variation to show any effect. Older adults, despite diminishing frequency of interaction due to health problems or major life transitions, report relatively high levels of perceived support due to their closer relationships with those who remain in their network or age-adjustments in expectations [38].

Measures of social relationships were not associated with cancer mortality in this study. A systematic review of observational studies showed inconsistent results; positive association mainly among those with cancer, and no association among those without cancer [39].

Strong associations between elevated depressive symptoms and deaths from all-causes, CVD, and non-CVD, non-cancer in our study agree with previous studies among older adults [40–42]. Behavioural mechanisms could be a potential explanation for the association between depression and all-cause and CVD mortality; depressed individuals are more likely to be physically inactive, to smoke, and to have unhealthy diet [43]. Indeed, our analysis showed that the significant association between elevated depressive symptoms and mortality attenuated after further adjustments for health-related behaviours. Moreover, it has been previously shown that depression without anxiety is associated with less help-seeking, which may in turn increase failure to adhere to treatment strategies [44]. In our study, elevated anxiety symptoms was not statistically significantly associated with mortality. While these results are consistent with some previous studies [45], they are contrary to other evidence [46, 47]. The methodological differences in ascertainment of anxiety could explain this; while we used a self-completed anxiety scale, others used a clinical diagnosis of anxiety [46, 47]. Of note, it has been previously shown that individuals with anxiety tend to seek more help [48], which could also explain the lack of association between elevated anxiety symptoms and mortality.

### Mediating role of social relationships and psychological distress on SES inequalities in mortality

Social relationships and psychological distress have been proposed as one of the underlying mechanisms of social inequalities in health [11]. However, very few studies have examined the mediating role of these factors on SES inequalities in mortality among older individuals [13, 16]. Stringhini et al. reported that social networks, positive/negative support, and loneliness explain cumulative SES inequalities in all-cause mortality by 11% and CVD-mortality by 8%, among 7846 British men and women aged ≥50 years [13] which is lower than what we found in our study (19% reduction in all-cause mortality and 17% reduction for CVD mortality). Possible explanations may include differences in population and methods. The only other study focusing on older people was in Taiwan (*n* = 4049, aged ≥60 years) and reported that emotional social support explained 25·8% of inequalities in all-cause mortality as assessed by educational level [16] which agrees with our findings. Another study among middle-aged individuals (35–55 years) from the UK, showed that measures of structural social support explained about one-third of the SES inequalities (assessed by occupational position) in all-cause and CVD mortality [8]. Two other studies with a wide range of age distributions (15–80 years) reported that social relationships explained 21 to 48% of the SES inequalities in all-cause mortality as assessed by education and income [12, 14].

Despite the considerable attention given to psychological mechanisms underlying SES inequalities in mortality [49], evidence is available from only two studies [15, 17]. One study among South Koreans aged ≥30 years reported that depression and perceived stress mediate SES inequalities in mortality by 11% [17]. The other among Dutch individuals aged 15–75 years reported that marital status, negative life events, medication use for anxiety, and depression explained 10% of income inequalities in CVD mortality [15]. Neither of these two studies conducted age-stratified analysis.

To our knowledge, our study represents the first evidence of a contribution of both social relationships and psychological distress to SES inequalities in all-cause and cause-specific mortality among older adults. The novel aspect in our analysis includes the identification of social relationships with greater precision than before, and the fact that we examined the associations between four individual indicators of SES as well as a cumulative SES score with not only all-cause mortality but also deaths from CVD, cancer, and non-CVD, non-cancer. Our results of 12 to 35% attenuation in SES inequalities in all-cause and cause-specific mortality strengthen the evidence that social relationships and psychological distress play an important role in older men’s health.

Multiple pathways have been proposed linking social relationships and psychological distress to health and mortality [50]. High levels of social support may be an effective buffer or modifier of stressful events. Moreover, social support could directly provide stability in life situation, irrespective of the presence of stress. Social relationships may influence health-related behaviours, such as smoking, physical inactivity, and alcohol consumption. A systematic review of longitudinal studies reported that depression and anxiety was associated with smoking itself as well as increased smoking frequency [51]. Psychological distress can interfere with adherence to treatment and medications, can affect coping strategies in the face of stress, as well as changes in inflammatory and cortisol responses [52]. Of note, social relationships and psychological distress may lie on the same causal pathway rather than being on separate pathways. For instance, low levels of social support might increase psychological distress, particularly after critical life events.

### Strengths and limitations

This study had the benefit of a long follow-up period and high-quality record linkage. We used both structural and functional social support as well as psychological distress, which better captures the different effects of psychosocial measures. Unlike previous studies, we examined the contribution of these factors to SES inequalities in both all-cause and cause-specific mortality. Moreover, the use of repeated measurements of social relationships and psychological distress allowed us to consider changes over time.

Our study also has some limitations. First, the structural and functional measures of social relationships that we used did not capture financial aid which is likely to moderate distress as people face financial strain [53]. Second, psychological distress was measured by self-report rather than by clinical diagnosis; the relation between clinical psychological distress and mortality as well as the mediating role of these factors may be stronger than using self-reported measures [40]. Both GDS and GAS are validated tools and widely used tools [24, 54], however, we cannot exclude the impact of the context of reporting (i.e. sex, population sampled), response bias (e.g. willingness to report, social desirability), and coping styles or trait (e.g. instrumentality, expressiveness) on reporting depressive or anxiety symptoms [55]. Third, the CHAMP study is a cohort of men aged ≥70 years; hence, the findings may not be applicable to women or younger adults or to other ethnicities. Fourth, due to the small number of deaths from cancer we were unable to examine associations by cancer sites. Fifth, the “change in estimate” method has some limitations such as model miss-specification and differential measurement error. Although we tried to minimise these limitations by testing for exposure-mediator interactions and controlling for mediator-outcome confounding, the size of estimates calculated from this method should be considered as approximate. Finally, although we adjusted for many relevant confounders and performed a series of sensitivity analysis, we cannot rule out the possibility of residual confounding due to unmeasured variables or covariates measured with error.

## Conclusion

A clear social gradient in social relationships and psychological distress was evident. Social relationships and depressive symptoms were associated with overall excess mortality and deaths from CVD and non-CVD, non-cancer. About 35% of the excess mortality among socioeconomically disadvantaged older men could be attributed to social relationships and psychological distress. These findings suggest that SES inequalities in mortality could at least partly be avoidable by implementing targeted interventions or policies with the aim of improving social relationships and phycological distress in disadvantaged older people.

## Supplementary information

**Additional file 1: Checklist 1.** STROBE Statement—Checklist of items that should be included in reports of *cohort studies*. **Supplementary Table 1.** Missing values of potential mediating factors and confounders throughout the follow-ups. **Supplementary Table 2.** Characteristics of the participants included and excluded from the analyses. **Supplementary Table 3.** Characteristics of participants by indicators of socioeconomic status at baseline, the CHAMP study. **Supplementary Table 4.** Associations of baseline individual socioeconomic status indicators and psychosocial measures, the CHAMP study. **Supplementary Table 5.** Associations of baseline cumulative socioeconomic status score and psychosocial measures, the CHAMP study-COMPLETE-CASE ANALAYSIS. **Supplementary Table 6.** Characteristics of participants by all-cause and cause-specific mortality status at baseline, the CHAMP study. **Supplementary Table 7.** Contribution of longitudinal psychosocial measures in explaining the association between individual indicators of socioeconomic status and all-cause and cause-specific mortality, the CHAMP study-IMPUTED. **Supplementary Figure 1.** Sample selection flow chart. **Supplementary Figure 2.** Associations between baseline measure of psychosocial measures and all-cause and cause-specific mortality, the CHAMP study- COMPLETE-CASE ANALAYSIS. **Supplementary Figure 3.** Contribution of longitudinal measure of psychosocial measures in explaining the association between socioeconomic status and all-cause and cause-specific mortality, the CHAMP study-SENSITIVITY ANALSYSIS AFTER EXCLUDING PARTICIPANTS WHO DIED IN THE FIRST TWO YEARS OF FOLLOW-UP. **Supplementary Figure 4.** Contribution of longitudinal measure of psychosocial measures in explaining the association between socioeconomic status and all-cause and cause-specific mortality, the CHAMP study-COMPLETE CASE ANALAYSIS.

## Data Availability

Some access restrictions apply to the data underlying this study’s findings. The original human ethics committee approval for the Concord Health and Ageing in Men Project (CHAMP) in 2004 did not allow for data to be sent outside Australia. Furthermore, the participants in CHAMP have not consented to their data being distributed beyond the CHAMP investigators and their associates. Qualified researchers may submit a request to the CHAMP Management Committee (robert.cumming@sydney.edu.au) and access will require additional ethics approval from the Sydney LHD HREC - CRGH, including considerations of privacy for data sharing.
