# Supplementary material for: Contribution of psychosocial factors to socioeconomic inequalities in mortality among older Australian men: a population-based cohort study
Source: Int J Equity Health. 2020 Oct 7;19:177. doi: 10.1186/s12939-020-01277-2 (PMC7539396; doi:10.1186/s12939-020-01277-2)
Supplement: Supplementary file 1 — Additional file 1: Checklist 1. STROBE Statement—Checklist of items that should be included in reports of cohort studies. Supplementary Table 1. Missing values of potential mediating factors and confounders throughout the follow-ups. Supplementary Table 2. Characteristics of the participants included and excluded from the analyses. Supplementary Table 3. Characteristics of participants by indicators of socioeconomic status at baseline, the CHAMP study. Supplementary Table 4. Associations of baseline individual socioeconomic status indicators and psychosocial measures, the CHAMP study. Supplementary Table 5. Associations of baseline cumulative socioeconomic status score and psychosocial measures, the CHAMP study-COMPLETE-CASE ANALAYSIS. Supplementary Table 6. Characteristics of participants by all-cause and cause-specific mortality status at baseline, the CHAMP study. Supplementary Table 7. Contribution of longitudinal psychosocial measures in explaining the association between individual indicators of socioeconomic status and all-cause and cause-specific mortality, the CHAMP study-IMPUTED. Supplementary Figure 1. Sample selection flow chart. Supplementary Figure 2. Associations between baseline measure of psychosocial measures and all-cause and cause-specific mortality, the CHAMP study- COMPLETE-CASE ANALAYSIS. Supplementary Figure 3. Contribution of longitudinal measure of psychosocial measures in explaining the association between socioeconomic status and all-cause and cause-specific mortality, the CHAMP study-SENSITIVITY ANALSYSIS AFTER EXCLUDING PARTICIPANTS WHO DIED IN THE FIRST TWO YEARS OF FOLLOW-UP. Supplementary Figure 4. Contribution of longitudinal measure of psychosocial measures in explaining the association between socioeconomic status and all-cause and cause-specific mortality, the CHAMP study-COMPLETE CASE ANALAYSIS. [file 12939_2020_1277_MOESM1_ESM.docx]

**Additional file**

**Title:** Contribution of psychosocial factors to socioeconomic inequalities in mortality among older Australian men: a population-based cohort study.

**Checklist 1.** STROBE Statement—Checklist of items that should be included in reports of *cohort studies*.

|  | Item No | Recommendation |
| --- | --- | --- |
| **Title and abstract** | 1 | (*a*) Indicate the study’s design with a commonly used term in the title or the abstract |
|  |  | (*b*) Provide in the abstract an informative and balanced summary of what was done and what was found |
| Introduction | | |
| Background/rationale | 2 | Explain the scientific background and rationale for the investigation being reported  **Introduction, paragraphs 1 and 2** |
| Objectives | 3 | State specific objectives, including any prespecified hypotheses  **Introduction, paragraph 3** |
| Methods | | |
| Study design | 4 | Present key elements of study design early in the paper  **Methods, paragraph 1** |
| Setting | 5 | Describe the setting, locations, and relevant dates, including periods of recruitment, exposure, follow-up, and data collection  **Methods, paragraph 1** |
| Participants | 6 | (*a*) Give the eligibility criteria, and the sources and methods of selection of participants. Describe methods of follow-up  **Methods (paragraph 1), Results (paragraph 1), figure S1, and table S1 & S2** |
|  |  | (*b*) For matched studies, give matching criteria and number of exposed and unexposed  **N/A** |
| Variables | 7 | Clearly define all outcomes, exposures, predictors, potential confounders, and effect modifiers. Give diagnostic criteria, if applicable  **Methods (Page 4 to 7)** |
| Data sources/ measurement | 8* | For each variable of interest, give sources of data and details of methods of assessment (measurement). Describe comparability of assessment methods if there is more than one group  **Methods (Page 4 to 7)** |
| Bias | 9 | Describe any efforts to address potential sources of bias  **Methods (Page 7 and 9 statistical analysis)** |
| Study size | 10 | Explain how the study size was arrived at  **Results (paragraph 1), figure S1 & table S1** |
| Quantitative variables | 11 | Explain how quantitative variables were handled in the analyses. If applicable, describe which groupings were chosen and why  **Methods (Page 4 to 9)** |
| Statistical methods | 12 | (*a*) Describe all statistical methods, including those used to control for confounding  **Methods (Statistical Analysis)** |
|  |  | (*b*) Describe any methods used to examine subgroups and interactions  **Methods (Statistical Analysis)** |
|  |  | (*c*) Explain how missing data were addressed  **Methods (Statistical analysis, paragraph 1), table S1** |
|  |  | (*d*) If applicable, explain how loss to follow-up was addressed  **Methods (Statistical analysis, paragraph 4)** |
|  |  | (*e*) Describe any sensitivity analyses  **Methods (Statistical analysis, last paragraph)** |
| Results | | |
| Participants | 13* | (a) Report numbers of individuals at each stage of study—eg numbers potentially eligible, examined for eligibility, confirmed eligible, included in the study, completing follow-up, and analysed  **Results, paragraph 1, Figure S1, and table S1 & S2** |
|  |  | (b) Give reasons for non-participation at each stage  **Methods, paragraph 1, figure S1, and table S1- we also referred to a previous study that described reasons for non-participation at each follow-up time of the CHAMP study.** |
|  |  | (c) Consider use of a flow diagram  **Figure 1 & table S1** |
| Descriptive data | 14* | (a) Give characteristics of study participants (eg demographic, clinical, social) and information on exposures and potential confounders  **Table 1 and table S2 & S3** |
|  |  | (b) Indicate number of participants with missing data for each variable of interest  **Table S1 and figure S1** |
|  |  | (c) Summarise follow-up time (eg, average and total amount)  **Results (paragraph 3) – this information was also added at the footnote of each tables** |
| Outcome data | 15* | Report numbers of outcome events or summary measures over time  **Results, paragraph 3 and table S6** |
| Main results | 16 | (*a*) Give unadjusted estimates and, if applicable, confounder-adjusted estimates and their precision (eg, 95% confidence interval). Make clear which confounders were adjusted for and why they were included  **Table 2, Figure 1 & 2, and supplementary Tables and Figures** |
|  |  | (*b*) Report category boundaries when continuous variables were categorized  **Method** |
|  |  | (*c*) If relevant, consider translating estimates of relative risk into absolute risk for a meaningful time period  **-** |
| Other analyses | 17 | Report other analyses done—eg analyses of subgroups and interactions, and sensitivity analyses  **Supplementary Tables (S4 & S5) and Figure S2 – S4** |
| Discussion | | |
| Key results | 18 | Summarise key results with reference to study objectives  **Discussion (paragraph 1)** |
| Limitations | 19 | Discuss limitations of the study, taking into account sources of potential bias or imprecision. Discuss both direction and magnitude of any potential bias  **Discussion (pages 16 & 17)** |
| Interpretation | 20 | Give a cautious overall interpretation of results considering objectives, limitations, multiplicity of analyses, results from similar studies, and other relevant evidence  **Discussion** |
| Generalisability | 21 | Discuss the generalisability (external validity) of the study results  **Discussion (Strengths and limitations)** |
| Other information | | |
| Funding | 22 | Give the source of funding and the role of the funders for the present study and, if applicable, for the original study on which the present article is based  **Funding (Page 20)** |

*Give information separately for exposed and unexposed groups.

**Note:** An Explanation and Elaboration article discusses each checklist item and gives methodological background and published examples of transparent reporting. The STROBE checklist is best used in conjunction with this article (freely available on the Web sites of PLoS Medicine at http://www.plosmedicine.org/, Annals of Internal Medicine at http://www.annals.org/, and Epidemiology at http://www.epidem.com/). Information on the STROBE Initiative is available at http://www.strobe-statement.org.

**Supplementary Table 1** Missing values of potential mediating factors and confounders throughout the follow-ups.

|  | Baseline | 2-year FU | | 5-year FU | 8-year FU |
| --- | --- | --- | --- | --- | --- |
|  | **Participants** |  |  |  |  |
|  | n=1522 | n=1239 | | n=882 | n=734 |
| Marital status | - | 4 | | 10 | 11 |
| Live alone | - | 4 | | 3 | 10 |
| Live with children | - | 4 | | 3 | 10 |
| Family support | - | 26 | | 3 | 14 |
| Non-family support | - | 28 | | 4 | 23 |
| Social interaction score | - | 13 | | 2 | 15 |
| Social satisfaction score | - | 9 | | 2 | 18 |
| Depressive symptoms | - | 11 | | 4 | 22 |
| Anxiety symptoms | - | 23 | | 2 | 0 |
| Alcohol consumption | 22 | 10 | | 6 | 44 |
| Smoking | 2 | 11 | | 2 | 17 |
| Physical activity | 3 | 8 | | 4 | 12 |
| Body mass index | 25 | 4 | | 23 | 65 |
| Self-rated health | 3 | 8 | | 2 | 12 |
|  |  | **Lost to follow-up & alive** | | | |
|  |  | n=169 | | n=172 | n=83 |

Abbreviation: FU, follow-up.

**Supplementary Table 2** Characteristics of the participants included and excluded from the analyses.

| Characteristics | Included  (n=1522) | Excluded  (n=183) | P-value ^a^ |
| --- | --- | --- | --- |
| Age, years | 77·4±5·5 | 77±5·4 | 0·31 |
| Age categories |  |  | 0·74 |
| 70-79 | 71·0 | 72·1 |  |
| 80+ | 29·0 | 27·9 |  |
| Country of birth |  |  | <0·001 |
| Australian-born | 51·5 | 35·5 |  |
| Other | 48·5 | 64·5 |  |
| **Structural and functional social support** |  |  |  |
| Marital status |  |  | 0·92 |
| Married/Defacto | 76·7 | 77·0 |  |
| Not married | 23·3 | 23·0 |  |
| Live alone | 18·7 | 18·6 | 0·64 |
| Live with children | 18·3 | 25·1 | 0·005 |
| No family support | 11·4 | 12·0 | 0·10 |
| No non-family support | 23·7 | 24·0 | 0·025 |
| Social interaction score | 6·0±1·3 | 5·8±1·4 | 0·040 |
| Social satisfaction |  |  | 0·25 |
| High | 62·7 | 50·8 |  |
| Low | 37·3 | 36·6 |  |
| **Psychological distress** |  |  |  |
| Depressive symptoms | 14·6 | 13·1 | 0·86 |
| Anxiety symptoms | 7·2 | 7·7 | 0·31 |
| **Health-related behaviours** |  |  |  |
| Alcohol consumption |  |  | 0·48 |
| Abstainer | 23·1 | 25·1 |  |
| Moderate drinkers | 67·8 | 65·0 |  |
| Heavy drinkers | 7·7 | 5·5 |  |
| Smoking |  |  | 0·43 |
| Non-smoker | 37·1 | 35·5 |  |
| Ex-smoker | 57·0 | 48·1 |  |
| Current smoker | 5·8 | 7·1 |  |
| Physical activity |  |  | 0·14 |
| Active | 75·4 | 63·4 |  |
| Inactive | 24·4 | 26·8 |  |
| Body mass index, kg/m^2^ | 27·8±3·9 | 28·3±4·8 | 0·11 |
| Body mass index categories |  |  | 0·79 |
| Underweight/normal | 24·2 | 23·3 |  |
| Overweight | 48·8 | 47·2 |  |
| Obese | 27·0 | 29·5 |  |
| Self-rated health |  |  | 0·59 |
| Good or excellent | 70·1 | 60·7 |  |
| Fair, poor, very poor | 29·9 | 28·4 |  |

Data are mean ± SD for continuous variables or percent for categorical variables, unless otherwise stated.

^a^ P-value calculated using Chi-square test for categorical variables and student’s t-test for continuous variables.

Due to some missing data, percentages do not always add to 100%.

**Supplementary Table 3** Characteristics of participants by indicators of socioeconomic status at baseline, the CHAMP study.

|  | Education | | |  | Occupation | | |  | Income | | |  | Housing tenure | |
| --- | --- | --- | --- | --- | --- | --- | --- | --- | --- | --- | --- | --- | --- | --- |
| Characteristics | High  (n=185) | Intermediate  (n=651) | Low  (n=686) |  | High  (n=469) | Intermediate  (n=574) | Low  (n=479) |  | High  (n=680) | Intermediate  (n=253) | Low  (n=589) |  | Owner  (n=1362) | Other  (n=160) |
| Age, years | 77·1±5·1 | 77·4±5·6 | 77·6±5·6 |  | 77·6±5·6 | 77·4±5·7 | 77·4±5·2 |  | 77·6±5·8 | 77·2±5·4 | 77·3±5·3 |  | 77·5±5·5 | 77·1±5·6 |
| Age categories |  |  |  |  |  |  |  |  |  |  |  |  |  |  |
| 70-79 (n=1080) | 71·4 | 71·9 | 70·0 |  | 69·5 | 72·6 | 70·4 |  | 67·1 | 74·7 | 73·9 |  | 70·1 | 78·1 |
| 80+ (n=442) | 28·6 | 28·1 | 30·0 |  | 30·5 | 27·4 | 29·6 |  | 32·9 | 25·3 | 26·1 |  | 29·9 | 21·9 |
| Country of birth |  |  |  |  |  |  |  |  |  |  |  |  |  |  |
| Australian-born (n=784) | 66·5 | 59·3 | 40·1 |  | 68·9 | 46·5 | 40·5 |  | 63·5 | 64·4 | 32·1 |  | 51·7 | 50·0 |
| Other (n=738) | 33·5 | 40·7 | 59·9 |  | 31·1 | 53·5 | 59·5 |  | 36·5 | 35·6 | 67·9 |  | 48·3 | 50·0 |
| **Structural and functional social support** |  |  |  |  |  |  |  |  |  |  |  |  |  |  |
| Marital status |  |  |  |  |  |  |  |  |  |  |  |  |  |  |
| Married/Defacto (n=1168) | 81·6 | 75·0 | 77·1 |  | 79·1 | 72·5 | 79·5 |  | 78·8 | 75·5 | 74·9 |  | 79·7 | 51·2 |
| Not married (n=354) | 18·4 | 25·0 | 22·9 |  | 20·9 | 27·5 | 20·5 |  | 21·2 | 24·5 | 25·1 |  | 20·3 | 48·8 |
| Live alone (n=284) | 16·8 | 20·4 | 17·5 |  | 15·8 | 15·8 | 16·9 |  | 17·5 | 17·5 | 18·8 |  | 16·9 | 16·9 |
| Live with children (n=279) | 10·8 | 14·7 | 23·8 |  | 14·5 | 19·0 | 21·3 |  | 14·6 | 15·0 | 24·1 |  | 17·9 | 21·9 |
| No family support (n=174) | 8·1 | 11·8 | 12·0 |  | 9·2 | 13·4 | 11·3 |  | 10·9 | 12·3 | 11·7 |  | 9·8 | 25·0 |
| No non-family support (n=361) | 15·7 | 21·7 | 27·8 |  | 15·4 | 26·3 | 28·8 |  | 21·3 | 15·8 | 29·9 |  | 22·8 | 31·3 |
| Social interaction score | 6·3±1·3 | 6·0±1·3 | 5·9±1·3 |  | 6·2±1·3 | 6±1·2 | 5·8±1·3 |  | 6·2±1·3 | 6·0±1·2 | 5·8±1·3 |  | 6·0±1·3 | 5·8±1·4 |
| Social satisfaction score | 19·8±1·9 | 19·4±2·5 | 19·2±2·3 |  | 19·8±2·1 | 19·2±2·5 | 19·1±2·4 |  | 19·6±2·1 | 19·5±2·2 | 19±2·6 |  | 19·5±2·1 | 18·1±3·5 |
| Social satisfaction |  |  |  |  |  |  |  |  |  |  |  |  |  |  |
| High (≥19, n=955) | 69·7 | 63·6 | 60·1 |  | 70·4 | 61·0 | 57·4 |  | 65·7 | 67·6 | 57·2 |  | 64·3 | 49·4 |
| Low (<19, n=567) | 30·3 | 36·4 | 39·9 |  | 29·6 | 39·0 | 42·6 |  | 34·3 | 32·4 | 42·8 |  | 35·7 | 50·6 |
| **Psychological distress** |  |  |  |  |  |  |  |  |  |  |  |  |  |  |
| Depressive symptoms (n=222) | 7·6 | 12·3 | 18·7 |  | 8·3 | 16·7 | 18·2 |  | 9·9 | 13·4 | 20·5 |  | 13·7 | 21·9 |
| Anxiety symptoms (n=109) | 4·9 | 6·9 | 8·0 |  | 6·6 | 6·6 | 8·4 |  | 6·6 | 7·9 | 7·5 |  | 6·6 | 11·9 |
| **Health-related behaviours** |  |  |  |  |  |  |  |  |  |  |  |  |  |  |
| Alcohol consumption |  |  |  |  |  |  |  |  |  |  |  |  |  |  |
| Abstainer (n=351) | 26·0 | 21·0 | 24·2 |  | 20·5 | 23·5 | 25·0 |  | 20·3 | 23·0 | 26·3 |  | 22·0 | 31·9 |
| Moderate drinkers (n=1032) | 65·4 | 70·8 | 65·6 |  | 69·3 | 67·6 | 66·6 |  | 70·3 | 65·6 | 66·2 |  | 68·9 | 58·7 |
| Heavy drinkers (n=117) | 7·0 | 7·2 | 8·3 |  | 8·7 | 8·0 | 6·3 |  | 8·4 | 10·3 | 5·8 |  | 7·6 | 8·1 |
| Missing (n=22) | 1·6 | 1·0 | 1·9 |  | 1·5 | 0·9 | 2·1 |  | 1·3 | 1·2 | 1·7 |  | 1·5 | 1·3 |
| Smoking |  |  |  |  |  |  |  |  |  |  |  |  |  |  |
| Non-smoker (n=564) | 49·2 | 39·0 | 31·9 |  | 43·7 | 35·2 | 32·8 |  | 41·2 | 36·4 | 32·6 |  | 36·9 | 38·1 |
| Ex-smoker (n=868) | 48·1 | 56·7 | 59·8 |  | 51·6 | 58·7 | 60·3 |  | 55·3 | 59·3 | 58·1 |  | 57·4 | 53·8 |
| Current smoker (n=88) | 2·7 | 4·3 | 8·0 |  | 4·5 | 6·1 | 6·7 |  | 3·4 | 3·9 | 9·3 |  | 5·5 | 8·1 |
| Missing (n=2) | 0·0 | 0·0 | 0·3 |  | 0·2 | 0·0 | 0·2 |  | 0·1 | 0·4 | 0·0 |  | 0·2 | 0·0 |
| Physical activity |  |  |  |  |  |  |  |  |  |  |  |  |  |  |
| Active (n=1147) | 73·0 | 79·6 | 72·0 |  | 74·6 | 76·5 | 75·1 |  | 76·9 | 74·3 | 74·0 |  | 77·5 | 57·5 |
| Inactive (n=372) | 26·5 | 20·4 | 27·7 |  | 25·2 | 23·5 | 24·9 |  | 22·6 | 25·7 | 26·0 |  | 22·4 | 41·9 |
| Missing (n=3) | 0·5 | 0·0 | 0·3 |  | 0·2 | 0·0 | 0·4 |  | 0·5 | 0·0 | 0·0 |  | 0·1 | 0·6 |
| Body mass index, kg/m^2^ | 26·8±3·8 | 27·7±3·8 | 28·1±4 |  | 27·3±3·8 | 27·7±3·8 | 28·3±4·2 |  | 27·7±3·8 | 27·3±3·9 | 28±4·1 |  | 27·8±3·9 | 27·2±4·5 |
| Body mass index categories |  |  |  |  |  |  |  |  |  |  |  |  |  |  |
| Underweight/normal (n=362) | 31·9 | 22·9 | 22·5 |  | 27·5 | 21·9 | 22·3 |  | 23·0 | 28·9 | 22·6 |  | 22·8 | 31·9 |
| Overweight (n=730) | 48·1 | 51·3 | 44·7 |  | 46·5 | 51·2 | 45·5 |  | 49·5 | 46·0 | 46·8 |  | 49·0 | 39·4 |
| Obese (n=405) | 18·4 | 24·0 | 31·3 |  | 24·5 | 25·3 | 30·3 |  | 26·5 | 23·3 | 28·2 |  | 26·6 | 26·2 |
| Missing (n=25) | 1·6 | 1·8 | 1·5 |  | 1·5 | 1·5 | 1·9 |  | 1·0 | 1·6 | 2·4 |  | 1·6 | 2·5 |
| Self-rated health |  |  |  |  |  |  |  |  |  |  |  |  |  |  |
| Good or excellent (n=1065) | 80·5 | 73·1 | 64·1 |  | 75·3 | 69·0 | 66·0 |  | 75·3 | 68·8 | 64·3 |  | 71·1 | 60·0 |
| Fair, poor, very poor (n=454) | 19·5 | 26·9 | 35·4 |  | 24·7 | 30·7 | 33·8 |  | 24·6 | 31·2 | 35·3 |  | 28·7 | 40·0 |
| Missing (n=3) | 0·0 | 0·0 | 0·5 |  | 0·0 | 0·3 | 0·2 |  | 0·1 | 0·0 | 0·4 |  | 0·2 | 0·0 |

N=1522. Data are mean ± SD for continuous variables or percent for categorical variables, unless otherwise stated.

Educational attainment was categorized as ‘high’ (university degree), ‘intermediate’ (trade, apprenticeship, certificate, or diploma), and ‘low’ (no post-school qualification); Occupational level was categorized as ‘high’ (higher professional and managers, lower professionals and managers, higher clerical services and sales workers), ‘intermediate’ (small employers and self-employed, farmers, lower supervisors and technicians), and ‘low’ (lower clerical, services, sales workers, skilled and unskilled workers); and source of income was categorized as ‘high’ (other sources of income only), ‘intermediate’ (reliant on a government pension plus other source of income), and ‘low’ (reliant on a government pension only).

**Supplementary Table 4** Associations of baseline individual socioeconomic status indicators and psychosocial measures, the CHAMP study.

|  | Model1 | Model2 | Model3 |
| --- | --- | --- | --- |
|  | OR (95% CI) ^a^ | OR (95% CI) ^b^ | OR (95% CI) ^c^ |
| **Education** (Lowest vs. highest) |  |  |  |
| **Structural social support** |  |  |  |
| Not married | 1·51 (0·99 to 2·31) | 1·53 (0·99 to 2·36) | 1·59 (1·03 to 2·45) |
| Living alone | 1·20 (0·77 to 1·87) | 1·20 (0·76 to 1·88) | 1·25 (0·79 to 1·97) |
| Live with children | 2·13 (1·29 to 3·53) | 2·18 (1·31 to 3·62) | 2·24 (1·34 to 3·73) |
| No family support | 1·69 (0·94 to 3·04) | 1·67 (0·93 to 3·01) | 1·59 (0·88 to 2·88) |
| No non-family support | 1·76 (1·14 to 2·73) | 1·82 (1·17 to 2·83) | 1·71 (1·10 to 2·68) |
| Social interaction (low vs. high) | 2·22 (1·49 to 3·31) | 2·35 (1·56 to 3·54) | 2·19 (1·45 to 3·30) |
| **Functional social support** |  |  |  |
| Social satisfaction (low vs. high) | 1·38 (0·97 to 1·97) | 1·41 (0·98 to 2·03) | 1·28 (0·89 to 1·85) |
| **Psychological measures** |  |  |  |
| Depressive symptoms (yes vs. no) | 2·45 (1·36 to 4·39) | 2·48 (1·36 to 4·53) | 1·86 (0·98 to 3·50) |
| Anxiety symptoms (yes vs. no) | 1·48 (0·82 to 2·65) | 1·48 (0·82 to 2·67) | 1·20 (0·66 to 2·19) |
|  |  |  |  |
| **Occupation** (Lowest vs. highest) |  |  |  |
| **Structural social support** |  |  |  |
| Not married | 1·22 (0·88 to 1·70) | 1·25 (0·89 to 1·74) | 1·27 (0·91 to 1·77) |
| Living alone | 1·36 (0·95 to 1·94) | 1·36 (0·95 to 1·95) | 1·39 (0·97 to 2·00) |
| Live with children | 1·27 (0·90 to 1·81) | 1·29 (0·91 to 1·83) | 1·30 (0·91 to 1·85) |
| No family support | 1·44 (0·93 to 2·22) | 1·40 (0·91 to 2·17) | 1·37 (0·88 to 2·12) |
| No non-family support | 1·96 (1·41 to 2·72) | 1·96 (1·40 to 2·72) | 1·91 (1·37 to 2·66) |
| Social interaction (low vs. high) | 1·74 (1·31 to 2·32) | 1·72 (1·28 to 2·30) | 1·67 (1·24 to 2·24) |
| **Functional social support** |  |  |  |
| Social satisfaction (low vs. high) | 1·61 (1·22 to 2·12) | 1·63 (1·23 to 2·16) | 1·57 (1·18 to 2·08) |
| **Psychological measures** |  |  |  |
| Depressive symptoms (yes vs. no) | 2·16 (1·43 to 3·26) | 2·11 (1·38 to 3·23) | 1·90 (1·22 to 2·97) |
| Anxiety symptoms (yes vs. no) | 1·37 (0·91 to 2·06) | 1·37 (0·90 to 2·08) | 1·26 (0·82 to 1·92) |
|  |  |  |  |
| **Income** (Lowest vs. highest) |  |  |  |
| **Structural social support** |  |  |  |
| Not married | 1·72 (1·29 to 2·29) | 1·71 (1·27 to 2·28) | 1·74 (1·30 to 2·33) |
| Living alone | 1·45 (1·06 to 1·97) | 1·43 (1·05 to 1·95) | 1·46 (1·07 to 2·00) |
| Live with children | 1·50 (1·11 to 2·02) | 1·47 (1·09 to 1·98) | 1·47 (1·09 to 1·98) |
| No family support | 1·27 (0·88 to 1·83) | 1·20 (0·83 to 1·74) | 1·17 (0·80 to 1·70) |
| No non-family support | 1·35 (1·03 to 1·76) | 1·31 (1·00 to 1·72) | 1·27 (0·97 to 1·67) |
| Social interaction (low vs. high) | 1·64 (1·28 to 2·10) | 1·53 (1·19 to 1·96) | 1·47 (1·14 to 1·89) |
| **Functional social support** |  |  |  |
| Social satisfaction (low vs. high) | 1·29 (1·02 to 1·64) | 1·26 (0·99 to 1·61) | 1·20 (0·94 to 1·53) |
| **Psychological measures** |  |  |  |
| Depressive symptoms (yes vs. no) | 2·10 (1·50 to 2·94) | 1·98 (1·40 to 2·81) | 1·83 (1·26 to 2·65) |
| Anxiety symptoms (yes vs. no) | 1·22 (0·84 to 1·76) | 1·19 (0·82 to 1·73) | 1·08 (0·74 to 1·59) |
|  |  |  |  |
| **Housing tenure** (Lowest vs. highest) |  |  |  |
| **Structural social support** |  |  |  |
| Not married | 4·29 (3·01 to 6·11) | 4·15 (2·89 to 5·96) | 4·25 (2·96 to 6·12) |
| Living alone | 2·73 (1·89 to 3·95) | 2·63 (1·80 to 3·83) | 2·68 (1·84 to 3·92) |
| Live with children | 1·26 (0·84 to 1·89) | 1·21 (0·80 to 1·84) | 1·22 (0·81 to 1·85) |
| No family support | 3·18 (2·12 to 4·76) | 2·97 (1·96 to 4·49) | 2·92 (1·93 to 4·42) |
| No non-family support | 1·56 (1·08 to 2·24) | 1·47 (1·01 to 2·13) | 1·43 (0·99 to 2·08) |
| Social interaction (low vs. high) | 1·54 (1·10 to 2·17) | 1·32 (0·93 to 1·88) | 1·29 (0·90 to 1·83) |
| **Functional social support** |  |  |  |
| Social satisfaction (low vs. high) | 1·85 (1·33 to 2·58) | 1·62 (1·15 to 2·27) | 1·57 (1·11 to 2·21) |
| **Psychological measures** |  |  |  |
| Depressive symptoms (yes vs. no) | 1·81 (1·20 to 2·74) | 1·37 (0·89 to 2·11) | 1·27 (0·79 to 2·02) |
| Anxiety symptoms (yes vs. no) | 1·56 (0·98 to 2·47) | 1·34 (0·83 to 2·16) | 1·27 (0·78 to 2·06) |

N=1522.

Cross-sectional association between individual socioeconomic status indicators and psychosocial measures were assessed by multivariable logistic regression.

The odd ratios of lowest versus highest socioeconomic status indicators are reported here.

^a^ Adjusted for age, age squared, and country of birth.

^b^ Further adjusted for health-related behaviours (alcohol consumption, smoking, and physical activity), and body mass index.

^c^ Further adjusted for self-rated health.

**Supplementary Table 5** Associations of baseline cumulative socioeconomic status score and psychosocial measures, the CHAMP study-COMPLETE-CASE ANALAYSIS.

|  | Model1 | Model2 | Model3 |
| --- | --- | --- | --- |
|  | OR (95% CI) ^a, b^ | OR (95% CI) ^a, c^ | OR (95% CI) ^a, d^ |
| **Structural social support** |  |  |  |
| Not married | 1·79 (1·31 to 2·45) | 1·76 (1·28 to 2·43) | 1·82 (1·32 to 2·51) |
| Living alone | 1·52 (1·09 to 2·14) | 1·49 (1·06 to 2·10) | 1·54 (1·09 to 2·17) |
| Live with children | 1·70 (1·24 to 2·35) | 1·70 (1·23 to 2·36) | 1·72 (1·24 to 2·39) |
| No family support | 1·43 (0·96 to 2·13) | 1·35 (0·90 to 2·03) | 1·32 (0·88 to 1·98) |
| No non-family support | 1·90 (1·42 to 2·55) | 1·88 (1·39 to 2·53) | 1·82 (1·35 to 2·45) |
| Social interaction (Low vs. high) | 1·66 (1·27 to 2·17) | 1·57 (1·19 to 2·07) | 1·50 (1·14 to 1·98) |
| **Functional social support** |  |  |  |
| Social satisfaction (Low vs. high) | 1·49 (1·15 to 1·93) | 1·45 (1·11 to 1·89) | 1·37 (1·05 to 1·79) |
| **Psychological measures** |  |  |  |
| Depressive symptoms (yes vs. no) | 2·41 (1·68 to 3·45) | 2·18 (1·50 to 3·17) | 1·89 (1·28 to 2·81) |
| Anxiety symptoms (yes vs. no) | 0·97 (0·59 to 1·59) | 0·90 (0·54 to 1·49) | 0·77 (0·46 to 1·29) |

N=1469.

Cross-sectional association between individual socioeconomic status indicators and psychosocial measures were assessed by multivariable logistic regression.

^a^ Cumulative socioeconomic status was entered as a 3-level categorical variable; the odd ratio of the lowest versus highest cumulative socioeconomic status are reported here.

^b^ Adjusted for age, age squared, and country of birth.

^c^Further adjusted for health-related behaviours (alcohol consumption, smoking, and physical activity), and body mass index.

^d^ Further adjusted for self-rated health.

**Supplementary Table 6** Characteristics of participants by all-cause and cause-specific mortality status at baseline, the CHAMP study.

|  | All-cause mortality | |  | Cause-specific mortality | | | | | | | |
| --- | --- | --- | --- | --- | --- | --- | --- | --- | --- | --- | --- |
|  |  |  |  | CVD | |  | Cancer | |  | Other ^a^ | |
| Characteristics | No  (n=745) | Yes  (n=777) |  | No  (n=1322) | Yes  (n=200) |  | No  (n=1315) | Yes  (n=207) |  | No  (n=1302) | Yes  (n=220) |
| Age, years | 75·2±4·2 | 79·6±5·8 |  | 76·8±5·2 | 81·8±5·5 |  | 77·3±5·6 | 78·1±5·3 |  | 76·9±5·3 | 80·5±6·1 |
| Age categories |  |  |  |  |  |  |  |  |  |  |  |
| 70-79 (n=1080) | 86·2 | 56·4 |  | 75·9 | 38·5 |  | 71·6 | 66·7 |  | 74·2 | 51·8 |
| 80+ (n=442) | 13·8 | 43·6 |  | 24·1 | 61·5 |  | 28·4 | 33·3 |  | 25·8 | 48·2 |
| Country of birth |  |  |  |  |  |  |  |  |  |  |  |
| Australian-born (n=784) | 47·7 | 55·2 |  | 50·8 | 56·5 |  | 51·3 | 52·7 |  | 50·4 | 58·2 |
| Other (n=738) | 52·3 | 44·8 |  | 49·2 | 43·5 |  | 48·7 | 47·3 |  | 49·6 | 41·8 |
| **Structural and functional social support** |  |  |  |  |  |  |  |  |  |  |  |
| Marital status |  |  |  |  |  |  |  |  |  |  |  |
| Married/Defacto (n=1168) | 83·5 | 70·3 |  | 79·0 | 62·0 |  | 76·7 | 76·8 |  | 77·7 | 70·9 |
| Not married (n=354) | 16·5 | 29·7 |  | 21·0 | 38·0 |  | 23·3 | 23·2 |  | 22·3 | 29·1 |
| Live alone (n=284) | 13·6 | 23·6 |  | 16·7 | 31·5 |  | 18·6 | 19·3 |  | 18·2 | 21·4 |
| Live with children (n=279) | 20·9 | 15·8 |  | 18·7 | 16·0 |  | 18·9 | 15·0 |  | 18·5 | 17·3 |
| No family support (n=174) | 8·2 | 14·5 |  | 32·1 | 46·5 |  | 33·7 | 35·7 |  | 31·4 | 49·1 |
| No non-family support (n=361) | 21·1 | 26·3 |  | 23·2 | 27·0 |  | 23·7 | 23·7 |  | 22·4 | 31·4 |
| Social interaction score | 6·3±1·3 | 5·8±1·3 |  | 6·1±1·3 | 5·7±1·3 |  | 6±1·3 | 6±1·4 |  | 6·1±1·3 | 5·5±1·3 |
| Social satisfaction score | 19·6±1·9 | 19·1±2·7 |  | 19·4±2·3 | 19±2·6 |  | 19·4±2·3 | 19·3±2·4 |  | 19·4±2·3 | 18·9±2·8 |
| Social satisfaction |  |  |  |  |  |  |  |  |  |  |  |
| High (n=955) |  |  |  |  |  |  |  |  |  |  |  |
| Low (n=567) | 74·4 | 58·0 |  | 67·9 | 53·5 |  | 66·3 | 64·3 |  | 28±3·9 | 50·9 |
| **Psychosocial factors** | 25·6 | 42·0 |  | 32·1 | 46·5 |  | 33·7 | 35·7 |  | 31·4 | 49·1 |
| Depressive symptoms (n=222) |  |  |  |  |  |  |  |  |  |  |  |
| Anxiety symptoms (n=109) | 7·9 | 21·0 |  | 13·1 | 24·5 |  | 13·9 | 18·8 |  | 12·5 | 26·8 |
| **Health-related behaviours** | 5·8 | 8·5 |  | 6·9 | 9·0 |  | 7·0 | 8·2 |  | 6·7 | 10·0 |
| Alcohol |  |  |  |  |  |  |  |  |  |  |  |
| Abstainer (n=351) | 19·9 | 26·1 |  | 22·4 | 27·5 |  | 22·8 | 24·6 |  | 21·8 | 30·4 |
| Moderate drinkers (n=1032) | 70·5 | 65·2 |  | 68·7 | 62·0 |  | 67·6 | 69·1 |  | 68·8 | 61·8 |
| Heavy drinkers (n=117) | 8·0 | 7·4 |  | 7·6 | 8·0 |  | 8·1 | 5·3 |  | 7·8 | 7·3 |
| Missing (n=22) | 1·6 | 1·3 |  | 1·3 | 2·5 |  | 1·5 | 1·0 |  | 1·6 | 0·5 |
| Smoking |  |  |  |  |  |  |  |  |  |  |  |
| Non-smoker (n=564) | 39·1 | 35·1 |  | 36·2 | 42·5 |  | 38·2 | 29·5 |  | 38·3 | 29·5 |
| Ex-smoker (n=868) | 56·6 | 57·4 |  | 57·9 | 51 |  | 56·3 | 61·8 |  | 56·0 | 63·2 |
| Current smoker (n=88) | 4·3 | 7·2 |  | 5·7 | 6·5 |  | 5·4 | 8·2 |  | 5·6 | 6·8 |
| Missing (n=2) | 0·0 | 0·3 |  | 0·1 | 0·0 |  | 1·0 | 0·5 |  | 1·0 | 0·5 |
| Physical activity |  |  |  |  |  |  |  |  |  |  |  |
| Active (n=1147) | 84·3 | 66·8 |  | 77·8 | 59·5 |  | 75·4 | 75·4 |  | 78·0 | 59·5 |
| Inactive (n=372) | 15·7 | 32·8 |  | 22·1 | 40·0 |  | 24·4 | 24·6 |  | 21·9 | 39·6 |
| Missing (n=3) | 0·0 | 0·4 |  | 0·1 | 0·5 |  | 0·2 | 0·0 |  | 0·1 | 0·9 |
| Body mass index, kg/m^2^ | 28·2±3·9 | 27·3±3·9 |  | 28±3·9 | 26·5±4 |  | 27·7±4 | 28·1±3·8 |  | 27·9±3·9 | 26·9±4 |
| Body mass index categories |  |  |  |  |  |  |  |  |  |  |  |
| Underweight/normal (n=362) | 19·1 | 28·3 |  | 22·0 | 35·5 |  | 24·0 | 22·2 |  | 22·4 | 32·3 |
| Overweight (n=730) | 49·8 | 46·2 |  | 48·4 | 45·0 |  | 48·2 | 46·4 |  | 48·8 | 42·7 |
| Obese (n=405) | 30·2 | 23·2 |  | 28·1 | 16·5 |  | 26·0 | 30·4 |  | 27·6 | 20·9 |
| Missing (n=25) | 0·9 | 2·3 |  | 1·5 | 3·0 |  | 1·8 | 1·0 |  | 1·2 | 4·1 |
| Self-rated health |  |  |  |  |  |  |  |  |  |  |  |
| Good or excellent (n=1065) | 77·6 | 62·7 |  | 71·4 | 60·5 |  | 70·7 | 65·2 |  | 71·8 | 59·1 |
| Fair, poor, very poor (n=454) | 22·3 | 37·0 |  | 28·4 | 39·5 |  | 29·0 | 34·8 |  | 28·0 | 40·5 |
| Missing (n=3) | 0·1 | 0·3 |  | 0·2 | 0·0 |  | 0·3 | 0·0 |  | 0·2 | 0·5 |

Abbreviations: CVD, cardiovascular disease; SES, socioeconomic status.

Data are mean ± SD for continuous variables or percent for categorical variables, unless otherwise stated.

^a^ Indicates non-cancer and non-cardiovascular disease mortality.

**Supplementary Table 7** Contribution of longitudinal psychosocial measures in explaining the association between individual indicators of socioeconomic status and all-cause and cause-specific mortality, the CHAMP study-IMPUTED.

|  | Education | | Occupation | | Source of income | | Housing tenure | |
| --- | --- | --- | --- | --- | --- | --- | --- | --- |
|  | HR (95% CI) ^a^ | %Attenuation ^b^ | HR (95% CI) ^a^ | %Attenuation ^b^ | HR (95% CI) ^a^ | % Attenuation ^b^ | HR (95% CI) ^a^ | % Attenuation ^b^ |
| **All-cause mortality** |  |  |  |  |  |  |  |  |
| Model 1  ^c^ | 1·45 (1·14 to 1·86) | - | 1·25 (1·04 to 1·51) | - | 1·49 (1·26 to 1·75) | - | 1·24 (1·00 to 1·55) | - |
| Model 1 + Structural measures | 1·36 (1·07 to 1·74) | 17 | 1·20 (1·00 to 1·44) | 18·5 | 1·43 (1·21 to 1·68) | 10·5 | 1·09 (0·87 to 1·37) | 60 |
| Model 1 + Functional measures | 1·42 (1·11 to 1·81) | 6 | 1·24 (1·03 to 1·49) | 6 | 1·45 (1·23 to 1·71) | 7 | 1·18 (0·95 to 1·47) | 24 |
| Model 1 + Structural +functional | 1·35 (1·06 to 1·73) | 19 | 1·20 (0·99 to 1·44) | 20 | 1·40 (1·19 to 1·66) | 14·5 | 1·06 (0·84 to 1·33) | 74 |
| Model 1 + Psychological distress | 1·35 (1·05 to 1·72) | 21 | 1·17 (0·97 to 1·41) | 30 | 1·39 (1·18 to 1·64) | 17 | 1·10 (0·89 to 1·37) | 55 |
| Model 1 + All psychosocial measures | 1·30 (1·01 to 1·66) | 30 | 1·16 (0·96 to 1·39) | 35 | 1·35 (1·15 to 1·60) | 24 | 1·00 (0·79 to 1·26) | 100 |
|  |  |  |  |  |  |  |  |  |
|  | SHR (95% CI) ^a^ | %Attenuation ^b^ | SHR (95% CI) ^a^ | %Attenuation^†^ | SHR (95% CI) ^a^ | %Attenuation ^b^ | SHR (95% CI) ^a^ | %Attenuation ^b^ |
| **CVD-mortality** |  |  |  |  |  |  |  |  |
| Model 1  ^c^ | 1·78 (1·08 to 2·95) | - | 1·49 (1·03 to 2·17) | - | 1·22 (0·88 to 1·68) | - | 1·01 (0·63 to 1·61) | - |
| Model 1 + Structural measures | 1·70 (1·03 to 2·82) | 8 | 1·45 (1·00 to 2·11) | 7 | 1·16 (0·84 to 1·61) | - | 0·86 (0·53 to 1·41) | - |
| Model 1 + Functional measures | 1·74 (1·05 to 2·88) | 4 | 1·49 (1·03 to 2·15) | 1·5 | 1·19 (0·86 to 1·66) | - | 0·97 (0·61 to 1·56) | - |
| Model 1 + Structural +functional | 1·67 (1·01 to 2·77) | 11 | 1·45 (1·00 to 2·11) | 7 | 1·15 (0·83 to 1·59) | - | 0·85 (0·52 to 1·39) | - |
| Model 1 + Psychological | 1·68 (1·01 to 2·79) | 10 | 1·44 (0·99 to 2·09) | 10 | 1·18 (0·84 to 1·64) | - | 0·95 (0·59 to 1·53) | - |
| Model 1 + All psychosocial measures | 1·62 (0·98 to 2·69) | 16·5 | 1·42 (0·97 to 2·07) | 13·5 | 1·13 (0·81 to 1·58) | - | 0·82 (0·50 to 1·36) | - |
| **Cancer mortality** |  |  |  |  |  |  |  |  |
| Model 1  ^c^ | 1·37 (0·83 to 2·25) | - | 1·34 (0·93 to 1·92) | - | 1·45 (1·05 to 1·98) | - | 0·96 (0·62 to 1·50) | - |
| Model 1 + Structural measures | 1·35 (0·82 to 2·22) | - | 1·33 (0·92 to 1·92) | - | 1·44 (1·05 to 1·99) | 1 | 0·93 (0·59 to 1·47) | - |
| Model 1 + Functional measures | 1·37 (0·84 to 2·26) | - | 1·34 (0·93 to 1·93) | - | 1·45 (1·06 to 1·99) | -1 | 0·97 (0·62 to 1·50) | - |
| Model 1 + Structural +functional | 1·36 (0·83 to 2·23) | - | 1·33 (0·92 to 1·92) | - | 1·45 (1·05 to 2·00) | -1 | 0·94 (0·60 to 1·48) | - |
| Model 1 + Psychological | 1·31 (0·80 to 2·15) | - | 1·29 (0·90 to 1·86) | - | 1·40 (1·02 to 1·92) | 9 | 0·91 (0·58 to 1·41) | - |
| Model 1 + All psychosocial measures | 1·32 (0·80 to 2·16) | - | 1·30 (0·90 to 1·88) | - | 1·42 (1·03 to 1·95) | 6 | 0·91 (0·58 to 1·44) | - |
| **Other mortality** |  |  |  |  |  |  |  |  |
| Model 1 ^c^ | 1·22 (0·78 to 1·93) | - | 1·03 (0·73 to 1·45) | - | 1·71 (1·26 to 2·32) | - | 1·61 (1·10 to 2·35) | - |
| Model 1 + Structural measures | 1·13 (0·71 to 1·80) | - | 0·98 (0·69 to 1·38) | - | 1·63 (1·20 to 2·20) | 9 | 1·43 (0·96 to 2·12) | 25·5 |
| Model 1 + Functional measures | 1·15 (0·73 to 1·82) | - | 0·99 (0·70 to 1·39) | - | 1·64 (1·21 to 2·23) | 8 | 1·47 (1·00 to 2·17) | 19 |
| Model 1 + Structural +functional | 1·10 (0·69 to 1·75) | - | 0·96 (0·68 to 1·35) | - | 1·59 (1·17 to 2·16) | 14 | 1·36 (0·91 to 2·05) | 35 |
| Model 1 + Psychological | 1·02 (0·64 to 1·63) | - | 0·91 (0·65 to 1·28) | - | 1·55 (1·13 to 2·11) | 18·5 | 1·38 (0·93 to 2·04) | 31 |
| Model 1 + All psychosocial measures | 0·99 (0·62 to 1·59) | - | 0·89 (0·63 to 1·25) | - | 1·49 (1·09 to 2·04) | 25 | 1·31 (0·87 to 1·99) | 43 |

Psychosocial measures assessed at baseline, first, second, and third follow-up.

^a^ Hazard ratios and sub-hazard ratios for lowest versus highest SES indicators are reported here.

^b^ Percent attenuation =100×(β_Model1_−β_Model1+ psychosocial measures(s)_)/ (β_Model1_), where β =log(Hazard ratio).

^c^ Adjusted for Adjusted for age, age squared, and country of birth.

Structural social support: marital status, live with children, family and non-family support, and social interaction score.

Functional social support: social satisfaction score.

Psychological distress: depressive and anxiety symptoms.

# **Supplementary Figure 1.** Sample selection flow chart.

Disagreement with mortality data linkage

(n=66, 3·9%)

Total original sample (n=1705)

Missing socioeconomic data (n=55, 3·2%)

Total analytic sample (n=1522, 89·3%)

Missing baseline psychosocial measures (n=62, 3·6%)

**Supplementary Figure 2** Associations between baseline measure of psychosocial measures and all-cause and cause-specific mortality, the CHAMP study- COMPLETE-CASE ANALAYSIS.

Psychosocial measures

**All-cause mortality**

**CVD mortality**

**Cancer mortality**

**Other mortality** ^a^

Hazard ratio (95% CI)

Sub-Hazard ratio (95% CI)

Sub-Hazard ratio (95% CI)

Sub-Hazard ratio (95% CI)

N= 1469.

^a^ Indicates non-cardiovascular disease and non-cancer mortality.

We used calendar year as the time scale, with survivors having a censoring date of 31 December 2017 (person years follow-up=13814) for all-cause mortality and with survivors having a censoring date of 31 December 2015 (person years follow-up=12180) for cause-specific mortality.

Model 1 adjusted for age, age squared, and country of birth.

Model 2 further adjusted for age, age squared, country of birth, health-related behaviours (alcohol consumption, smoking, and physical activity), and BMI.

Model 3 further adjusted self-rated health.

**Supplementary Figure 3** Contribution of longitudinal measure of psychosocial measures in explaining the association between socioeconomic status and all-cause and cause-specific mortality, the CHAMP study-SENSITIVITY ANALSYSIS AFTER EXCLUDING PARTICIPANTS WHO DIED IN THE FIRST TWO YEARS OF FOLLOW-UP.

SHR (95% CI) ^c^

Adjustment

Adjustment

% Attenuation

HR (95% CI) ^c^

**All-cause mortality** ^a^

**CVD mortality** ^b^

**Cancer mortality** ^b^

**Other mortality** ^b^

SHR (95% CI) ^c^

% Attenuation

Adjustment

Adjustment

% Attenuation

SHR (95% CI) ^c^

% Attenuation

^d^

^d^

^d^

^d^

Abbreviations: HR, hazard ratio; SHR, sub-hazard ratio.

N=1434 after excluding 88 participants who died the first two years of follow-up. There were 689, 170, 169, and 200 deaths attributable to all-cause, CVD, cancer, and non-cancer, non-CVD mortality.

^a^ We used calendar year as the time scale, with survivors having a censoring date of 31 December 2017 (person years follow-up=13667).

^b^ We used calendar year as the time scale, with survivors having a censoring date of 31 December 2015 (person years follow-up=12031).

^c^ Hazard ratios and sub-hazard ratios for lowest versus highest cumulative socioeconomic status are reported here.

Percent attenuation =100×(β_Model1_−β_Model1+psychosocial measures(s)_)/ (β_Model1_), where β =log(Hazard ratio).

^d^ Adjusted for age, age squared, and country of birth.

Structural social support: marital status, live with children, family and non-family support, and social interaction score.

Functional social support: social satisfaction score.

Psychological distress: depressive and anxiety symptoms.

**Supplementary Figure 4** Contribution of longitudinal measure of psychosocial measures in explaining the association between socioeconomic status and all-cause and cause-specific mortality, the CHAMP study-COMPLETE CASE ANALAYSIS.

SHR (95% CI) ^c^

Adjustment

Adjustment

% Attenuation

HR (95% CI) ^c^

**All-cause mortality** ^a^

**CVD mortality** ^b^

**Cancer mortality** ^b^

**Other mortality** ^b^

SHR (95% CI) ^c^

% Attenuation

Adjustment

Adjustment

% Attenuation

SHR (95% CI) ^c^

% Attenuation

^d^

^d^

^d^

^d^

Abbreviations: HR, hazard ratio; SHR, sub-hazard ratio.

N=860. There were 400, 106, 133, and 100 deaths attributable to all-cause, CVD, cancer, and non-cancer, non-CVD mortality.

^a^ We used calendar year as the time scale, with survivors having a censoring date of 31 December 2017 (person years follow-up=7667).

^b^ We used calendar year as the time scale, with survivors having a censoring date of 31 December 2015 (person years follow-up=6685).

^c^ Hazard ratios and sub-hazard ratios for lowest versus highest cumulative socioeconomic status are reported here.

Percent attenuation =100×(β_Model1_−β_Model1+psychosocial measures(s)_)/ (β_Model1_), where β =log(Hazard ratio).

^d^ Adjusted for Adjusted for age, age squared, and country of birth.

Structural social support: marital status, live with children, family and non-family support, and social interaction score.

Functional social support: social satisfaction score.

Psychological distress: depressive and anxiety symptoms.
